# Specificity and Effector Functions of Human RSV-Specific IgG from Bovine Milk

**DOI:** 10.1371/journal.pone.0112047

**Published:** 2014-11-06

**Authors:** Gerco den Hartog, Shamir Jacobino, Louis Bont, Linda Cox, Laurien H. Ulfman, Jeanette H. W. Leusen, R. J. Joost van Neerven

**Affiliations:** 1 Laboratory of Translational Immunology, Immunotherapy group, UMC Utrecht, Utrecht, The Netherlands; 2 Cell Biology and Immunology, Wageningen University, Wageningen, The Netherlands; 3 Department of Pediatrics, UMC Utrecht, Utrecht, The Netherlands; 4 Bioceros, Utrecht, The Netherlands; 5 FrieslandCampina, Amersfoort, The Netherlands; University of Tennessee Health Science Center, United States of America

## Abstract

**Background:**

Respiratory syncytial virus (RSV) infection is the second most important cause of death in the first year of life, and early RSV infections are associated with the development of asthma. Breastfeeding and serum IgG have been shown to protect against RSV infection. Yet, many infants depend on bovine milk-based nutrition, which at present lacks intact immunoglobulins.

**Objective:**

To investigate whether IgG purified from bovine milk (bIgG) can modulate immune responses against human RSV.

**Methods:**

ELISAs were performed to analyse binding of bIgG to human respiratory pathogens. bIgG or hRSV was coated to plates to assess dose-dependent binding of bIgG to human Fcγ receptors (FcγR) or bIgG-mediated binding of myeloid cells to hRSV respectively. *S. Epidermidis* and RSV were used to test bIgG-mediated binding and internalisation of pathogens by myeloid cells. Finally, the ability of bIgG to neutralise infection of HEp2 cells by hRSV was evaluated.

**Results:**

bIgG recognised human RSV, influenza haemagglutinin and *Haemophilus influenza*. bIgG bound to FcγRII on neutrophils, monocytes and macrophages, but not to FcγRI and FcγRIII, and could bind simultaneously to hRSV and human FcγRII on neutrophils. In addition, human neutrophils and dendritic cells internalised pathogens that were opsonised with bIgG. Finally, bIgG could prevent infection of HEp2 cells by hRSV.

**Conclusions:**

The data presented here show that bIgG binds to hRSV and other human respiratory pathogens and induces effector functions through binding to human FcγRII on phagocytes. Thus bovine IgG may contribute to immune protection against RSV.

## Introduction

Respiratory syncytial virus (RSV) infection is a major cause of death in the first year of life, with especially high mortality rates in African and Asian countries [1], [2], [3]. Upper respiratory tract infections (URTI) with RSV generally cause relatively mild disease that does not require treatment. These infections can progress, however, into lower respiratory tract infections (LRTI) and cause severe disease, especially in pre-mature infants. RSV can also cause recurrent infection of the upper respiratory tract.

An important complication of upper respiratory infections is middle ear inflammation and-related deafness [4], [5], and RSV infections are associated with the development of asthma at a later age [6], [7], [8].

Other common causes for respiratory tract infections are *Haemophilus influenzae* type b (Hib), *parainfluenza virus, rhinovirus* and *influenza virus* [9], [10], [11].

Protective immunity against respiratory pathogens like influenza and RSV is mediated by IgG and IgA [12], [13], [14], [15], [16]. RSV-specific serum IgG levels in neonates are inversely associated with increased prevalence of RSV infections [17], [18], and breastfeeding reduces the incidence and severity of RSV infection [19], [20]. In addition, the levels of anti-influenza IgA in breast milk correlates with decreased frequency of respiratory illness with fever [16]. These findings indicate that pathogen-specific antibodies are crucial for protection against respiratory infections, and that orally ingested immunoglobulins (like breastmilk-derived IgA) may contribute to immunity to airway infections.

Human and bovine milk contain high levels of immunoglobulins, which are important for protecting the infant from infections with bacteria and viruses [21], [22]. Cross-species activity between human and bovine immune-related milk proteins has been reported before [23], [24], and cow’s milk contains bovine IgG (bIgG) that binds to gastrointestinal pathogens that also infect humans, such as *Shigella flexneri, Escherichia coli, Clostridium difficile, Streptococcus mutants, Cryptosporidium parvum, Helicobacter pylori,* and rotavirus [25].

At present there is, however, no information on binding of bovine IgG to human respiratory viruses.

Most infant nutrition is bovine milk-based, but lacks intact bIgG as a result of heat treatment during processing. To investigate if bIgG would be a useful ingredient in these formulas, the aim of the present study was to investigate the specificity and functional relevance of bIgG against RSV and other human respiratory pathogens, the ability of bIgG to bind to human Fcγ receptors, and the induction of effector functions in human myeloid cells.

## Materials and Methods

### Bovine milk samples and preparation of bIgG

bIgG was purified from commercially available bovine colostrum (Colostrum 35% IgG, Reflex Nutrition, Bristol, UK) using an AFFI-T™ column (Kem-en-Tec) followed by a protein G column (5 ml; Amersham). bIgG was eluted with 0.1 M glycine-HCl pH 2.7 elution buffer and neutralised with 1 M Tris-HCl pH 9.0, followed by dialysation against PBS and sterilisation (0.2 µm filter). Fresh milk and colostrum samples were supplied by FrieslandCampina (the Netherlands).

### Detection of pathogen-specific IgG

Maxisorb ELISA-plates (Nunc) were coated with 0.5–2 µg/ml pathogen antigens, derived from human vaccines (Influvac 2012/2013 (0.5 µg/ml), Abbott Biologicals), Act-HIB (Sanofi pasteur MSD (0.5 µg/ml)), DTP (1/200, Nederland vaccine instituut), or 25 µl inactivated RSV A2 or rhinovirus (kindly provided by Prof. S Johnston, Imperial College London, UK). Plates were blocked with 0.5% gelatine/PBS, washed (0.05% tween-20/PBS), and samples were added and titrated. Plates were washed four times and 1/2000 HRP-conjugated sheep anti bovine IgG1 (Abd Serotec, Kidlington, UK) or 1/6000 anti-human IgG (Jackson ImmunoResearch, West Grove, PA, USA) were used as detector antibody. Plates were washed extensively and developed with TMB and read at 450 nm. For inhibition ELISAs purified IgG from human plasma (Intravenous Immunoglobulin, pooled from >1000 donors, or IVIg) (Sanquin blood supply, The Netherlands) or bIgG equivalent to 167 µg/ml was pre-incubated at room temperature with twice the amount used for coating of hRSV or vaccine. Binding to F protein of bRSV was analysed by a commercial ELISA, according to the manufacturer’s descriptions (Bio-X Diagnostics, Jemelle, Belgium).

### Generation of RSV extracts

RSV A2 was added to HEp2 cells (IMDM with L-glutamin, HEPES and 1% FSC) and incubated for six days. Cells were lysed (0.5% NP-40) [26]) and used for ELISAs.

Also PEG-precipitated RSV lysate was prepared. Slowly ice cold 50% PEG6000 in 150 mM NaCl, 1 mM EDTA and 6.1 g/L Tris was added to RSV-infected Hep2 cells while stirring (end concentration PEG was 10%). The mixture was stirred at 4°C for three hours. The PEG-precipitated virus mixture was centrifuged (30 min, 4000 rpm at 4°C). Supernatant was removed and the pellet taken up in 10% sucrose and stored in N2.

### Isolation and culture of human myeloid cells

Ethical approval of the use of blood samples was obtained from the institutional review boards the ‘Medical Ethical Committee’ of the UMC Utrecht, The Netherlands and Sanquin Blood Supply, The Netherlands. Review and approval was obtained prior to the experiments were conducted and are in accordance with the declaration of Helsinki. Donors provided written informed consent and the blood samples were used anonymously. Blood was obtained at the UMC Utrecht (collected in heparin vacutainers (BD Biosciences) or buffy coats (Sanquin blood supply, The Netherlands) were diluted 1∶1 in PBS. Diluted blood was layered on top of a Histopaque-ficoll gradient and centrifuged for 25 min, 1500 rpm, slow acceleration and without brake. PBMCs and PMNs were harvested and washed in RPMI. Red blood cells were lysed with ammonium buffer (pH 7, UMC Utrecht pharmacy) for 10 minutes on ice.

Monocytes were purified using CD14 microbeads (Myltenyi biotec, Bergisch Gladbach, Germany), according the manufacturers prescriptions, with the following modifications: cells were incubated in half the volume of MACS buffer and CD14 beads, and incubated for 30 minutes on a roller at 4°C. Monocytes were cultured in RPMI containing 10% FCS. For differentiation to macrophages, monocytes were cultured overnight in the presence of 10 ng/ml IFN-γ (Peprotech, Rocky Hill, NJ, USA). Monocyte-derived DCs (moDC) were differentiated for five days under the influence of 50 ng/ml GM-CSF (Peprotech).

### Binding of bIgG to cells expressing FcγR

96 wells maxisorb plates (Nunc) were coated with Ig in 0.1 M NaHPO_4_, pH 9 overnight (4°C). Cells were incubated with 5 µM calcein AM (Invitrogen) in PBS (PMNs were labelled in HEPES [27]) for 30 min at 37°C (PMNs for 20 min), washed with RPMI containing 0.2% gelatin. 1.5*10^5^ calcein-labelled cells were added, centrifuged (500 rpm, 5 min) and allowed to attach for 45 min at 37°C (10 min for PMN) in RPMI containing 0.2% gelatin. Initial fluorescence and fluorescence after several washing steps was recorded (excitation 485 nm, emission 527 nm, ThermoFisher Scientific Fluoroskan Ascent FL). For FcγR-specific inhibition of binding of calcein-labelled cells 2 or 5 µg/ml Fab fragments of clone 3G8 (FcγRIII) or IV.3 (FcγRII) were used. Stable FcγRI-transfected IIA1.6 cells were previously shown to bind to human IgG but not bIgG and were therefore not included in this study [28].

### Pathogen-specific Ig cell-binding assay

96 wells maxisorb plates were coated with PEG-precipitated RSV at 4°C overnight. Plates were washed (PBS) and blocked with 1% gelatine/PBS (1 hour 37°C) and washing three times (PBS-tween). Bovine or human IgG (or buffer) was allowed to bind to RSV for 3 hours at room temperature. Plates were washed 3 times followed by washing once with RPMI containing 0.2% gelatine. The assay was continued as described under ‘Binding of IgG to human FcyR’.

### FcγR-mediated internalisation assay

8*10^5^ CFU/ml FITC-labelled *S. epidermidis* was incubated with 20 µl of varying concentrations of IVIg or bIgG overnight at 4°C. Twenty µl of pre-cooled cells (1.5*10^5^) were added and incubated with the bacteria on ice for 30 min. Plates were washed and incubated at 37°C or 4°C (45 min. macrophages, 20 min moDCs) in 150 µl RPMI. Cells were washed and stained with 1/200 biotinylated anti FITC antibodies (Southern Biotec), followed by washing and 1/100 streptavidin APC (eBiosciences). Cells were washed and fixed with 1% PFA/PBS and analysed on FACS Canto II. All washing steps were performed three times with ice-cold FACS buffer and a cooled centrifuge at 1500 rpm for 5 minutes.

### GFP-RSV internalisation assay

Renilla-GFP RSV (1*10∧5 plaque forming units (PFU)) was pre-incubated for 1 hour at 37°C with 40 µl RPMI (10% FCS and 1% pen/strep) in the presence or absence of antibodies and then cooled for 15 min on ice. Pre-cooled PMN (1.5*10∧5 cells) and 40 µl RSV-Ig mixture was added and allowed to bind for 15 min in 96-wells plates. Plates were washed and incubated for 15 min at 37°C in pre-warmed RPMI. After washing, cells were treated with trypsin for 10 min on ice to remove extracellular RSV. Cells were incubated twice (30 sec) with acid medium (RPMI pH 2.5, 0.1% FCS) to remove FcR bound RSV-Ig, with a 1 min centrifugation step (1500 rpm) in between and afterwards. Finally, cells were washed and fixed with 1% PFA in PBS and GFP expression was analysed by flow cytometry.

### RSV neutralisation assay

HEp2 cells (5*10∧4 cells) cultured in IMDM (10% FCS, 1% pen/strep) were seeded overnight in flat-bottom 96-wells plates. Renilla-GFP RSV (1*10∧5 PFU) was pre-incubated for 1 hour at 37°C with 100 µl medium (1% FCS) in the presence or absence of antibodies and then added to the cells. Cells were trypsinised after 18–24 hour incubation and GFP expression was analysed by flow cytometry.

## Results

### Binding of bIgG to human respiratory tract pathogens

**Figure 1:**
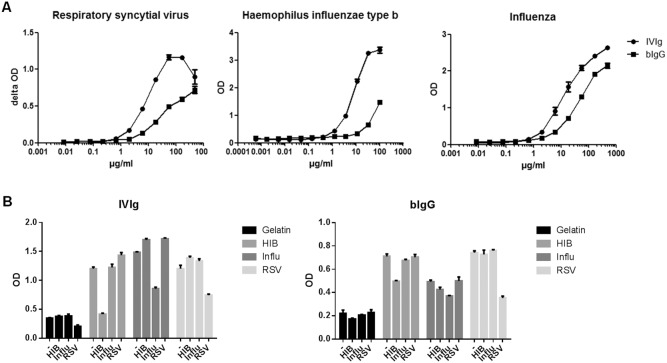
bIgG binds to human airway pathogens. **A**) RSV, influenza or *Haemophilus influenzae* type b was coated in ELISA plates and human (IVIg) or bovine (bIg) IgG was added in different concentrations (x-axes in µg/ml). Mean OD or delta OD (RSV) values and S.E.M. are shown of triplicate measurements. **B**) Inhibition of binding of IVIg (left) or bIg (right) to vaccines by pre-incubating the Ig-samples (167 µg/ml) with the antigen. Horizontal text below graphs indicates vaccine used for coating, whereas diagonal text indicates the vaccine used for pre-incubation; ‘−’ indicates pre-incubation with medium. Mean and S.E.M. of triplicate measurements are shown.

To study binding of purified human plasma IgG (IVIg) and bovine colostrum IgG (bIgG) to human RSV (hRSV) lysate of HEp2 cells (control) or RSV infected HEp2 cells was coated to an ELISA plate. Dose-dependent binding of bIgG to RSV (>2 SD above background) was observed at concentrations starting at 1.8 µg/ml (Figure 1A).

IVIg and bIgG also bound to flu and Hib antigens (Figure 1A). Pathogen-specific bIgG could be detected at levels up to 11 µg/ml. Compared to bIgG, less IVIg was required to detect the pathogens. Binding of bIgG to rhinovirus was only detectable at very high IgG levels (data not shown). hRSV, HIB and influvac-specific IgG could also be detected in pooled bovine milk samples (not shown).

To test the specificity of the bIgG-binding to human respiratory pathogens, inhibition ELISAs were performed. These experiments confirmed the specificity of the binding of bIgG to RSV, influenza and Hib (Figure 1B). However, binding of bIgG to flu could also be partly inhibited by Hib, suggesting the presence of cross reactive epitopes.

### Binding of bIgG to human FcγRs

**Figure 2:**
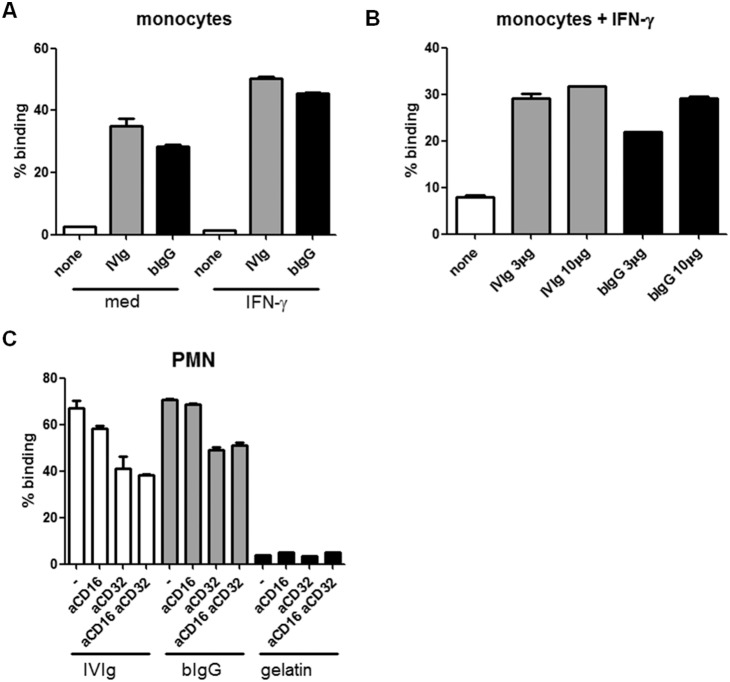
bIgG binds to human FcγRII. **A**) IgG was immobilised on 96 wells ELISA plates. Monocytes were incubated overnight in the absence or presence of 10 ng/ml IFN-γ. **B**) Different concentrations of IgG were coated and IFN-γ stimulated monocytes were added. **C**) Binding of PMNs to 10 µg/ml human (IVIg) and bovine IgG (bIg). PMNs were incubated for 10 minutes in the absence (–) or presence of 3 µg/ml Fabs recognizing FcγRIII (CD16) or FcγRII (CD32). As negative control, plates were coated with gelatin (none) instead of Ig. Binding of bIgG by monocytes and PMNs was observed for three different donors. For all panels binding of cells after five washes is shown and expressed as percentage of initial. Mean and S.E.M. is shown of triplicate measurements.

To study binding of bIgG to human FcγRs, monocytes were incubated overnight in the presence or absence of IFN-γ to enhance FcγR expression, and exposed to plate-bound bIgG. Human monocytes of 5 out of 5 donors bound to immobilised bIgG, as well as to IVIg, which was increased by pre-incubating the cells with IFN-γ (Figure 2A). Binding of monocytes to bIgG was dose-dependent and comparable or slightly lower compared to human IVIg (Figure 2B).

Also binding of PMN to bIgG was evaluated. PMNs of 4/5 donors bound to IVIg as well as to bIgG (Figure 2C).

To identify which receptor was involved in binding of PMN to bIgG, unstimulated PMN which lack FcγRI [29], were incubated with FcγRII (Fab)- and FcγRIII (F(ab)2)-specific blocking mAb prior to addition of the cells to the Ig-coated plates (Figure 2C). Pre-incubation with FcγRII-blocking Fabs reduced binding of PMN to bIgG of all three donors tested. Binding of PMN in both the presence and absence of FcγRII-specific Fabs was comparable for IVIg and bIgG, although some donor-specific differences were observed. Pre-incubation of PMN with FcγRIII-specific Fabs resulted in slightly decreased binding of IVIg, but not of bIgG.

### Binding of macrophages and PMN to RSV-specific Ig

**Figure 3:**
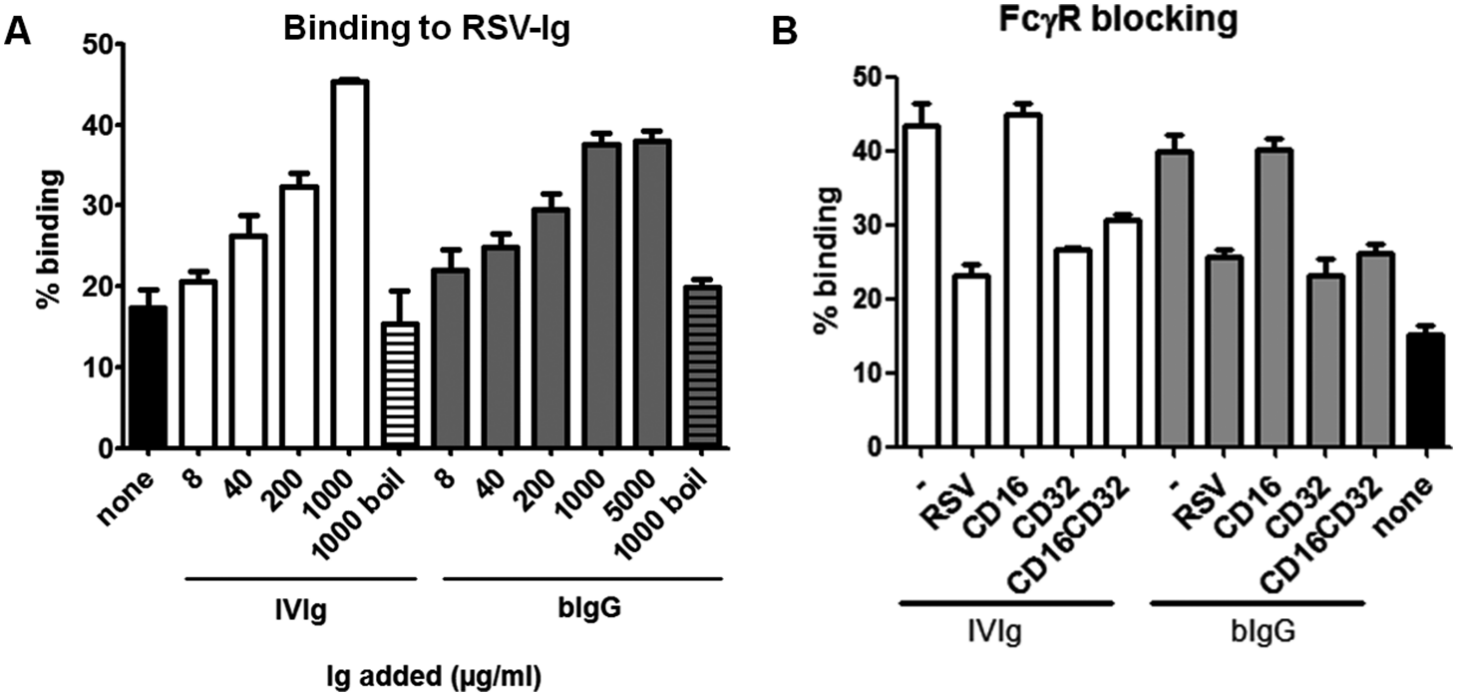
Bovine IgG simultaneously binds to human RSV and human FcγRII. Plates were coated with PEG-precipitated RSV, grown in HEp2 cells. **A**) Varying concentrations of human (IVIg) or bovine (bIgG) Ig were added, followed by incubation with calcein-labelled macrophages. A representative example of four donors tested is shown. No binding of cells was observed when Ig was inactivated prior to addition to the plate, or when Ig was added to gelatine coated plates (data not shown). **B**) 1000 µg/ml IVIg or bIgG was added to RSV-coated plates. PMNs were pre-incubated with medium, and/or anti FcγRII (CD32) or FcγRIII (CD16) Fabs. Independent experiments were performed with three donors. Graphs show mean and S.E.M. of triplicate measurements.

To study whether human phagocytes can bind to bIgG that has captured RSV, an experiment was performed requiring bIgG to simultaneously bind to RSV immobilised on a plate and human macrophages (Figure 3A). Binding of cells to RSV-specific IVIg or bIgG was dose-dependent and lost when IgG was (boiled prior to addition to the plate). No binding was observed when plates were coated with gelatin instead of RSV (data not shown). Bovine RSV-specific IgG was also able to bind simultaneously to RSV and PMN (Figure 3B).

Next, PMN were pre-incubated with anti FcγRII and/or anti FcγRIII Fabs prior to addition to RSV-bound Ig. Pre-incubation of PMN with anti FcγRII, but not anti FcγRIII Fabs, reduced binding of PMN to RSV-specific IVIg and bIgG (Figure 3B). Pre-incubation of Ig with RSV completely inhibited cell-binding, confirming that cell-binding in this assay is indeed RSV-specific.

### bIgG induces FcγR-mediated phagocytosis

In addition to extracellular binding of pathogens, internalisation is required for efficient clearance of pathogens and efficient induction of memory T cells. FITC-labelled bacteria (*S. epidermidis*) were used as model for bIgG-mediated internalisation of pathogens by macrophages and moDCs. To discriminate between extracellular bound bacteria and internalised bacteria, APC-conjugated antibodies recognising FITC were used.

**Figure 4:**
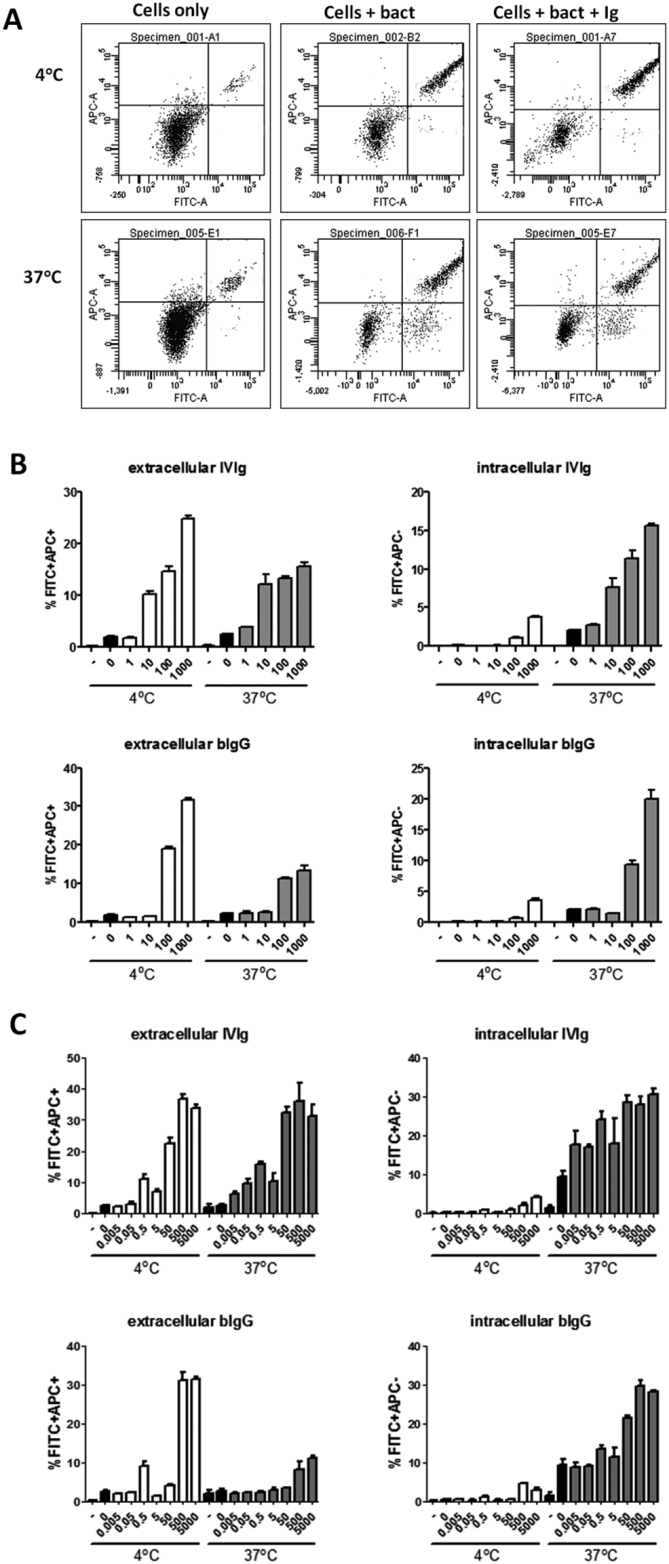
bIg-mediated binding and phagocytosis of *S. epidermidis* by IFN-γ-stimulated monocytes and GM-CSF-differentiated moDCs. FITC-labelled bacteria were opsonised or not with human (IVIg) or bovine (bIgG) IgG. Subsequently cells were allowed to bind to opsonised bacteria and incubated at 4°C (negative control) or 37°C degrees and stained with APC-conjugated antibodies recognizing FITC. Extracellular bacteria were defined as FITC+APC+ and intracellular bacteria as FITC+APC−. Extracellular bacteria can be observed at both 4°C and 37°C incubated cells, whereas intracellular bacteria are only present in cells incubated at 37°C. A) Example of FACS dot plot and gating strategy. B and C) Percentage of IFN-γ conditioned monocytes (B) and moDCs (C) with extracellular (left) and intracellular (right) bacteria of IVIg (top) and bIgG (bottom) incubated at 4°C or 37°C (indicated at x-axes). Black bars indicate medium (–) or bacteria alone without Ig (0). X-axes show µg/ml Ig used for opsonisation of bacteria. Mean and S.E.M. of triplicate measurements are shown of one out of three donors tested.

Medium, *S. epidermidis* left non-opsonised or opsonised with different concentrations of IVIg or bIgG were added to macrophages. Cells were allowed to bind to bacteria at 4°C, washed and incubated at 37°C (allowing internalisation) or 4°C (allowing binding, but not internalisation) for 45 minutes. Subsequently, cells were stained with APC anti FITC. Both IVIg and bIgG enhanced the binding of bacteria to macrophages, indicated by increased percentages of cells that are FITC positive (Figures 4A and B). Also internalisation (FITC^+^APC^−^) of bacteria was enhanced by IVIg and bIgG. The increased binding and internalisation was dose-dependent, and only observed for cells incubated at 37°C.

Similarly, bIgG enhanced internalisation of *S. epidermidis* by moDCs of three other donors (Figure 4C). These data show that bIgG is able to enhance binding as well as internalisation of pathogens by human immune cells.

### Binding and internalisation of soluble hRSV by human PMNs

**Figure 5:**
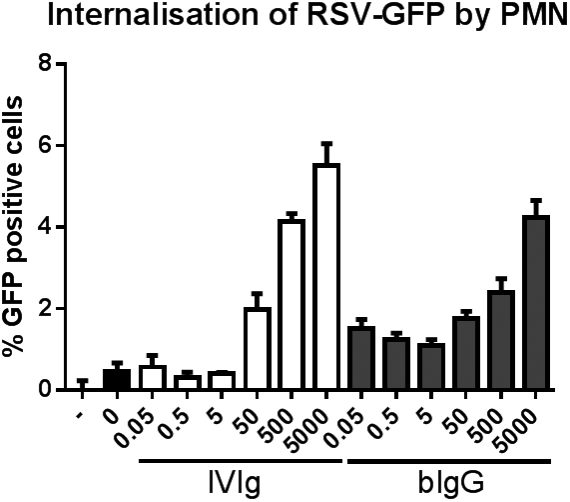
Bovine Ig enhances internalisation of hRSV by hPMN. GFP-renilla expressing RSV was pre-incubated with medium in the presence or absence of IVIg or bIgG and allowed to bind to PMN at 4°C. Subsequently, cells were incubated at 37°C and thereafter treated with trypsin and acid to remove extracellular RSV. Cells were then washed and analysed by flow cytometry for the percentage of GFP+ cells. GFP+ cells were tested in the absence of RSV (–), in the presence of RSV but absence of Ig (0) and in the presence of IVIg or bIgG (µg/ml). Mean and S.E.M. of triplicate measurements of one out of five donors tested are shown.

PMN, like macrophages, play a crucial role in the elimination of opsonised pathogens. To investigate whether pathogen-opsonisation by bIgG can enhance internalisation by human PMNs GFP-labelled hRSV was opsonised with bIgG. In the absence of antibodies little internalisation of hRSV by human PMN was observed, measured as the percentage of GFP positive cells by flow cytometry (Figure 5). By contrast, pre-incubation of hRSV with both IVIg and bIgG resulted in a dose-dependent increase of hRSV internalisation. While IVIg was more effective than bIgG at the two highest concentrations tested, bIgG was particularly more effective at lower concentrations.

### Neutralisation of HEp2 infection by RSV

**Figure 6:**
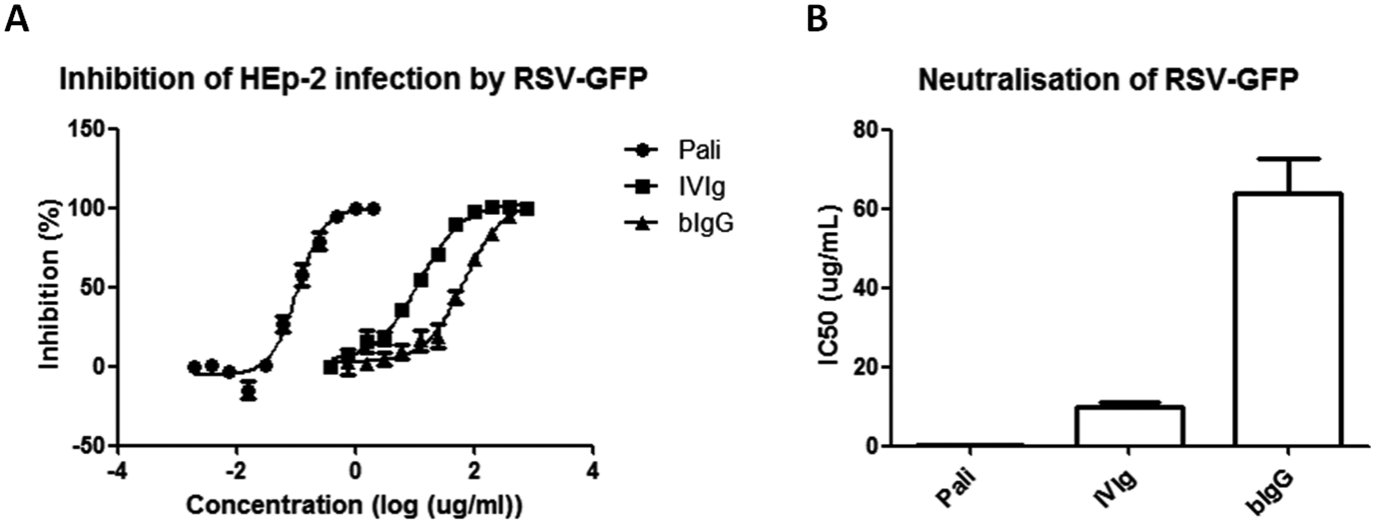
Neutralisation of infection of HEp-2 cells by human RSV. **A**) 5*10∧4 HEp-2 cells were seeded overnight in flat bottom 96-wells plates and infected the next day with 1*10∧5 PFU RSV-GFP which was pre-incubated with different concentrations of bovine (bIgG) or human (IVIg) IgG or monoclonal F-protein-specific palivizumab. GFP intensity was determined by flow cytometry as a measure for HEp2 cell infection by RSV-GFP. For the calculation of the inhibition percentage the MFI of uninfected cells was set to 100% and the MFI of infected cells without Ab incubation to 0%. Mean and S.E.M. of three independent experiments is shown. **B**) IC50 values for neutralisation of RSV-GFP by palivizumab, IVIg and bIgG are shown.

Pre-term birth children receive intravenous palivizumab to prevent RSV infection [30]. The F-protein epitope recognised by palivizumab seems to be conserved between human and bovine RSV as palivizumab also recognises the F protein of bovine RSV (data not shown). Therefore, like palivizumab, bIgG might be able to prevent infection with hRSV. To this aim GFP-renilla-RSV was pre-incubated with bIgG, IVIg or palivizumab and added to HEp2 cells. After 18–24 hours incubation, cells were harvested and analysed for GFP expression by flow cytometry. Both IVIg and bIgG dose-dependently neutralised RSV, although 6.4 times more bIgG compared to IVIg was needed to inhibit HEp2 cell infection by RSV (IC50: 64 and 10 µg/ml, respectively) (Figure 6B).

## Discussion

Pathogen-specific immunoglobulins are supplied to newborns through breastfeeding. These immunoglobulins help to protect the neonate against infections by pathogenic bacteria and viruses. Neonates, especially those born pre-maturely, are relatively vulnerable to pathogenic infections in the respiratory tract [2]. We aimed to study whether intact bovine milk-derived immunoglobulins can modulate immune responses to respiratory pathogens. The data presented here show that bIgG recognises several pathogens that are known to cause infections in the respiratory tract of infants. In addition, bIgG bound to human FcγRII, and enhance phagocytosis of pathogens by human myeloid immune cells. Bovine IgG was shown to bind to human RSV, and could even prevent infection of HEp2 cells by human RSV *in vitro*.

Cows are susceptible to pathogens like bovine RSV, and transfer bovine RSV-specific antibodies into colostrum and mature milk after delivery [31], [32]. Those observations suggest that cow’s indeed seem to develop neutralising antibodies against bRSV, that cross-react with hRSV. It is relevant to note that even though significant levels of bIgG specific for human respiratory pathogens were detected, the cows were not immunised against human pathogens. However, it is common practice to vaccinate calves against bovine RSV around weaning when the maternal-derived IgG levels are decreasing. Our data suggest that for RSV, protecting the calf against bRSV through immunisation may give rise to milk that has the potential to be protective against bovine as well as human RSV.

bIgG was also able to neutralise infection of HEp2 cells with hRSV. Compared to palivizumab, a humanized anti-RSV therapeutic mAb, and IV-Ig, a significantly higher concentration of bIgG was required for neutralisation of infection of HEp2 cells by hRSV. Palivizumab is often given prophylactically and therefore is present at the moment when hRSV is encountered by the neonate. Direct neutralization of hRSV by orally deposited bIgG on mucosal surfaces is possible, but not very likely. Activated T and B cells recirculate throughout the body and home to different tissues. Tissue specific homing explains why oral administration of antigen-specific IgG leads to increased presence of memory T and B cells in other organs like the lungs and the breasts [33], [34], [35], [36], [37]. Likewise, passive oral vaccination has been shown to induce active immune responses in the gastro-intestinal (GI) tract, but also in the upper airways and in breast tissue [38]. Therefore, bIgG consumption may induce adaptive immunity against respiratory pathogens in a similar manner. Dietary bIgG encounters swallowed respiratory pathogens in the GI tract or in the tonsillar crypts in Waldeyer’s ring, forming immune-complexes that efficiently bind to DCs in the tissue [39], [40].

As bIgG can bind to RSV, this may lead to viral uptake through FcγR-mediated phagocytosis and, therefore, may facilitate an enhanced T-cell response through binding to FcγRII, as reported by Gosselin for human IgG [41]. The interaction of bIgG with hFcγRII and internalisation of bIgG immune-complexes has been observed for multiple donors, indicating it is a robust phenomenon (Table S1).

As shown in Figure 3, bIgG could bind simultaneously to hRSV and FcγRII on human innate immune cells, and moDCs could internalise bIgG-coated hRSV, which is needed for antigen presentation. This confirms earlier findings that demonstrated binding of bIgG to human FcγRII expressed on myeloid cells [42]. Cross-linking of FcγRII by DCs causes NFκß activation and maturation of DCs [39]. FcγRII-mediated antigen uptake and presentation, as well as DC maturation is crucial for activation and differentiation of T cells. As a result bIgG may facilitate adaptive T cell responses. This can thus result in long-lasting protection against airway pathogens via the T-cell dependent induction of pathogen-specific (IgA) antibodies in the upper airways.

Our current findings are based on various in vitro assays and require in vivo studies to show formally demonstrate that like Ig from breast milk, bIgG can assist the development of immune memory responses. Another aspect that needs further study is how effective bIgG is in inducing protective immunity in immunocompromised individuals, like children suffering from recurrent asthma caused by viral infections, and in newborns in whom the immune system has not yet matured. Therefore, inclusion of intact bIgG in infant nutrition may contribute to the induction of adaptive immunity against airway infections. Interestingly, several epidemiological studies have linked the consumption of milk to a reduced incidence of asthma [43], [44], [45], [46]. This effect was later shown to be associated with the consumption of raw milk, and the detectability of intact milk proteins [47]. As RSV infections may predispose infants to develop asthma at a later age [6], [7], [8], we would like to speculate that there is a possibility that the effect we describe here for RSV-specific bovine antibodies may contribute to the effect of raw milk consumption on asthma development.

Although nutritional intervention studies are needed to formally demonstrate if dietary bIgG can contribute to protection against respiratory pathogens, and possibly also against the development of asthma, the *in vitro* data reported here support this concept.

## Supporting Information

Table S1 Overview of donors used for various assays.
(DOCX)
