# Supplementary material for: Specificity and Effector Functions of Human RSV-Specific IgG from Bovine Milk
Source: PLoS One. 2014 Nov 6;9(11):e112047. doi: 10.1371/journal.pone.0112047 (PMC4222812; doi:10.1371/journal.pone.0112047)
Supplement: Table S1 — Overview of donors used for various assays. (DOCX) [file pone.0112047.s001.docx]

**Table S1. Overview of donors used for various assays**

|  | **FcγR binding** | | **RSV and FcR binding** | | | **Internalisation** | | **total** |
| --- | --- | --- | --- | --- | --- | --- | --- | --- |
|  | ***IVIg*** | ***bIgG*** | ***IVIg*** | ***bIgG*** | ***IVIg*** | | ***bIgG*** |  |
| monocyte | 2 | 2 |  |  |  | |  | 2 |
| PMN | 3 | 3 | 13 | 9 |  | |  | 16 |
| macrophage | 3 | 3 |  |  | 5 | | 5 | 6 |
| DC |  |  |  |  | 3 | | 3 | 3 |
| Unique donors | 7 | (100%) | 13 | (69%) | 7 | | (100%) | 27 (85%) |

Numbers in the table indicate the number of donors with positive test results (all experiments included IVIg and bIgG and are shown in the table). Percentage between brackets in bottom row indicate the percentage of donors binding to bIgG compared to IVIg
